# Supporting undergraduate students’ developing water literacy during a global pandemic: a longitudinal study

**DOI:** 10.1186/s43031-022-00049-y

**Published:** 2022-03-07

**Authors:** Silvia-Jessica Mostacedo-Marasovic, Diane Lally, Destini N. Petitt, Holly White, Cory Forbes

**Affiliations:** 1 University of Texas at Arlington, Texas Arlington, USA; 2 Industry, Lexington, Kentucky USA; 3 University of North Carolina at Charlotte, Charlotte, North Carolina USA; 4 University of Maine, Orono, Maine USA

**Keywords:** Water literacy, Water-based models, Course effectiveness, COVID-19

## Abstract

To prepare students to address water-related challenges, undergraduate STEM education must provide them with opportunities to learn and reason about water issues. Water in Society is an introductory-level, innovative, and interdisciplinary undergraduate course offered annually at a large midwestern university. The course focuses on both disciplinary concepts and civic engagement, and is designed around a variety of interactive, research-based practices to support students’ learning, engagement with authentic data, scientific models and modeling, and collaboration and learning among peers. This study aims to evaluate, “*how have student outcomes and perceptions changed over five years of the course?”.* The results are based on data from students (*n =* 326) in five consecutive years of the course, during which time the course transitioned from a face-to-face model to fully asynchronous online model due, in part, to impacts of the COVID-19 pandemic. The particularly rapid and abrupt transition between 2020 and 2021 in response to COVID-19 led to many course changes, including modes of communication between instructors and students and opportunities for collaboration. Here, multiple measures are used to evaluate students’ learning about water concepts, model-based reasoning about socio-hydrologic systems, and perceptions of the course across all five years. By the end of each iteration of the course, students improved their knowledge of hydrologic concepts, independent of the course format or other student-level variables. However, results also show that students’ performance on complex socio-hydrologic systems modeling tasks, as well as their overall satisfaction with the course, decreased in Year 5 when the course was fully online. Results provide insight into efforts to move undergraduate STEM courses online and specific evidence of the COVID-19 pandemic’s impacts on undergraduate STEM teaching and learning about water.

**Supplementary Information:**

The online version contains supplementary material available at 10.1186/s43031-022-00049-y.

## Introduction

Water is a critical component supporting all life on Earth. It is interrelated with every human activity, such as agriculture, power generation, industrial production, mining, public and domestic supply, human health, education, and culture. Between 1900 and 2010, global water use increased by a six-fold factor; and, with population growth, economic development, and changes in consumption patterns, it continues to increase at a rate of 1% per year (Aquastat, n.d. in UNESCO & UN Water, 2020). In conjunction with climate change, this adds increasing pressure on both the quantity and quality of surface and groundwater resources (UNESCO & UN Water, 2020; IPCC, 2021). Furthermore, with human populations rapidly expanding, tensions around water resource access and use are likely to intensify. As a result, the world faces many socio-hydrologic issues (SHIs) that are complex in nature and involve both scientific and non-scientific dimensions. Efforts to conserve and effectively manage Earth’s water resources require decision-making based on scientific evidence, as well as social, economic, and policy considerations to ensure water security within complex socio-hydrological systems. Teaching and learning about water in an array of contexts, including undergraduate classrooms, is therefore pivotal to help future water researchers, practitioners, and global citizens develop water literacy, which involves hydrologic knowledge, skills, attitudes, and behaviors, to enable sustainable water management practices (King et al., 2012; McCarroll & Hamann, 2020; Su et al., 2011).

Despite evidence of the value of water and its importance across different STEM disciplines (King et al., 2012), research has found that there are many challenges to cultivating water literacy through education. At the undergraduate level, students may have inaccurate or incomplete understandings about water, the water cycle, or water systems (Arthurs & Elwonger, 2018; Attari et al., 2017; Cardak, 2009; Sibley et al., 2007), which can persist into adulthood (Duda et al., 2005; Williams et al., 2009) and need opportunities to develop understanding of environmental principles within a framework that foregrounds coupled human-natural systems (King et al., 2012). Many innovative educational strategies have been applied to improve or enhance undergraduate water education, including technological approaches (Habib et al., 2012; Li & Liu, 2003; Williams et al., 2009), developing and evaluating content material (White et al., 2017), and creating interdisciplinary courses (Willermet et al., 2013). While these studies highlight positive impacts of these approaches on undergraduate water education, they also illustrate persistent challenges in supporting undergraduate students’ learning about water and development of water literacy.

To contribute to these broader efforts, we have developed, offered, and evaluated the undergraduate Water in Society course at a large midwestern university since 2017. Water in Society is a 3-credit hour, introductory, interdisciplinary course offered to students from STEM and non-STEM majors that has served over *n =* 326 students in the past five years. The course has a dual focus on students 1) developing understanding of introductory-level core hydrologic concepts and 2) using that knowledge to analyze, reason, and make decisions about water-related issues. In an earlier publication, we described the origins and initial design of the course (Forbes et al., 2018). Since then, the course has maintained a consistent focus on a core set of innovative research-based practices to support students’ learning and engagement with authentic data, scientific models and modeling, and the use of evidence to make informed decisions about SHIs. However, there have also been changes to the course over time, particularly a shift from a more traditional, instructor-driven course to a hybrid, flipped, and more student-centered course design; and, most recently, to a fully online and asynchronous course in response to COVID-19. Since 2017, we have extensively studied these aspects of the course as it has evolved (Lally et al., 2020; Lally & Forbes., 2019,  2020; Owens et al., 2020; White & Forbes, 2021), contributing to the broader body of research on undergraduate water education. Collectively, these studies have provided insight into the use of modeling and visualization tools to help students reason about hydrologic concepts and engage in decision-making about SHIs, as well as how the design of the course can support these outcomes. These studies build upon and contribute to insights from our broader body of research on undergraduate water education (Petitt & Forbes, 2019;  Sabel et al., 2017).

Despite this multi-year discipline-based research effort, we have not yet conducted a comprehensive evaluation of the course outcomes. While prior studies have focused on specific course elements within one or two years, they have not sought to account for a broader range of outcomes across all five years as the course has evolved, or how these outcomes may have been impacted by the global COVID-19 pandemic. The present study aims to provide a systematized account of the five years of the course, in which we evaluate how students’ outcomes and perceptions have changed over five years, including the spring 2020 and 2021 semesters, in which the COVID-19 crisis necessitated a shift to virtual learning. It provides evidence about undergraduate water education from an interdisciplinary and holistic perspective, efforts to continue improving the course, and informs other education researchers and practitioners about strategies that could be used to improve the design of courses related with water resources. The research question guiding this study is, “*how have the students’ outcomes and perceptions changed over five years of the course?”.*

### Undergraduate water education

Teaching and learning about water remain an important priority within undergraduate education. Among hydrologists, for example, there has been a growing recognition of the need for innovative educational approaches to prepare the next generation of water scientists (Wagener et al., 2012). This has led to a growing body of work illustrating innovative approaches in undergraduate water science courses (Habib et al., 2012; Halvorson & Westcoat, 2002; Kingston et al., 2012; Li & Liu, 2003; Smith et al., 2006; Thompson et al., 2012). In addition, there is an emphasis on cultivating water literacy among the population to support science-based water-related decision-making (Attari et al., 2017; Covitt et al., 2009; Johnson & Courter, 2020; King et al., 2012; McCarroll & Hamann, 2020; Su et al., 2011). As King et al. (2012) observe, “Appreciating that the subject matter of hydrology is embedded in a larger context of causes and effects, which includes human decision-making and generates complex system behaviors, is a primary step in reframing hydrology education” (pg. 4025). In this sense, addressing the complexity of water-related challenges, requires that students from STEM and Non-STEM majors to develop an understanding about different natural and human dimensions of water. Within this context, some work has explored interdisciplinary courses for both STEM majors (including those in water sciences) and non-majors (Covitt et al., 2009; Forbes et al., 2018; Smith et al., 2006; Willermet et al., 2013; Williams et al., 2009). All of these efforts share a commitment to the idea that individuals must learn to apply disciplinary knowledge to the social, economic, legal, and political dimensions of water-related issues, or *water literacy.*

However, research has shown that water literacy in the United States remains underdeveloped. Students across the K-16 continuum, including undergraduate students, demonstrate an array of scientifically inaccurate conceptions about water and water systems (Arthurs & Elwonger, 2018; Attari et al., 2017; Cardak, 2009; Covitt et al., 2009; Halvorson & Westcoat, 2002; Sibley et al., 2007). For example, students may not fully grasp water vapor as a component of air (Cardak, 2009; Sibley et al., 2007), groundwater stocks and flows (Arthurs & Elwonger, 2018; Cardak, 2009), the relation between the built environment and water (Attari et al., 2017; Covitt et al., 2009), and interactions between natural and human dimensions of complex water systems (Halvorson & Westcoat, 2002). Prior research documents also observed that many commonly observed ideas students articulate (i.e., groundwater existing as underground reservoirs, evaporation occurring only from large bodies of surface water, the impact of Earth’s oceans on weather and climate, and the vast amount of Earth’s freshwater used for agricultural production), often underemphasize less-directly-observable components of water systems, such as soil moisture, groundwater, and water vapor, in their reasoning about hydrologic stocks and flows. Some evidence suggests these alternative ideas may carry over into adulthood (Duda et al., 2005). Collectively, while these findings highlight many characteristics of water and water systems that learners do grasp, they also identify specific areas in which water literacy can be enhanced, including through undergraduate education.

### Reform-based, model-centric undergraduate teaching and learning

To support their learning about water and development of water literacy, undergraduate students should use computational, simulation-based models in classroom settings which, when combined with other pedagogical approaches, can support learners to develop a more comprehensive understanding of hydrology (AghaKouchak et al., 2013; Habib et al., 2012; Merwade & Ruddell, 2012). Models are an important tool with which to support students’ learning about complex systems, including water. Modeling helps students engage with otherwise inaccessible phenomena and develop skills, including explaining ideas, making connections between the real world and scientific concepts, evaluation of models and ideas, and metacognitive processes. While scientific models can take a variety of forms (visual representations, physical models, computer simulations, analogies, etc.) (Lally & Forbes, 2019; Merwade & Ruddell, 2012), here we focus on data-driven, computer-based computational models for water systems, tools commonly used by hydrologists. With these models at their disposal, students can explore multiple hypotheses, develop policies, and quickly run multiple scenarios involving hydrologic phenomena and socio-hydrologic systems (Gunn et al., 2002; Williams et al., 2009; Zigic & Lemckert, 2007).

Enhancing undergraduate water education using models, simulations, and visualizations harnesses the benefits of reform-based instruction, including active learning, to interact with authentic data for complex analysis and decision-making (AghaKouchak et al., 2013; Attari et al., 2017; Gunn et al., 2002; King et al., 2012; Lally & Forbes, 2019, 2020; Merwade & Ruddell, 2012; Sibley et al., 2007; Zigic & Lemckert, 2007). Effective, model-centric instructional strategies align with best practices in undergraduate STEM instruction (Handelsman et al., 2004) and innovative teaching strategies to positively affect student outcomes (Gunn et al., 2002). Effective teaching and learning in STEM disciplines has evolved from traditional approaches that prioritize one-directional information transmission through instructor-led lectures and ‘canned’ activities with stringent, pre-determined outcomes. Instead, contemporary perspectives emphasize active learning, a process in which instructors and students co-participate in higher order thinking and co-create knowledge and meaning (Handelsman et al., 2004). Prior research has demonstrated the positive impact of reform-based pedagogy and active learning in undergraduate courses. Active learning involves an array of alternative classroom structures, including small and large group discussions, problem solving, practicing, questioning, and feedback, through which students, create, analyze, and evaluate evidence and knowledge claims for natural phenomena (Habib et al., 2012; Halvorson & Westcoat, 2002; Kingston et al., 2012; Li & Liu, 2003; Smith et al., 2006; Thompson et al., 2012; White et al., 2017; Willermet et al., 2013). This compelling evidence underlies current emphases on use of active learning strategies and principles of effective STEM instruction in undergraduate STEM courses.

## Methods

### Study design and context

This convergent mixed-methods evaluation study is based on student data from five consecutive years of the Water in Society course, offered annually in the spring semester at a large, R1 institution in the U.S. Midwest (Lally et al., 2020; Lally & Forbes, 2019, 2020; Owens et al., 2020; White & Forbes, 2021). Over these five years, the course has gradually shifted from a lecture-based to a student-centered format, with varying types and degrees of active-learning opportunities. Furthermore, while the course content has remained largely the same, the modality of the course has shifted from traditional face-to-face (2017) to a flipped hybrid model (2018 and 2019), a split flipped and fully online modality (2020), to a fully asynchronous online model (2021). Students’ interactions with the content, instructors, and other students changed from a higher interaction between students and instructors and their peers in 2017 to a predominant degree of student-content interaction, followed by student-instructor interaction, and less student-student interaction in 2021 (Li et al., 2016) (Table 1).

**Table 1:** Gradual shift in learning approaches

Approach	2017	2018	2019	2020	2021
Lecture-based	85%	70%	50%	30%	15%
Active learning	15%	30%	50%	70%	85%
Mode: *(1) In person; (2) online; (3) synchronous; (4) asynchronous*	(1)	(1)	(1)	(1), (2), (3)	(2), (4)
Priority of course interactions: *(1) Student-instructor; (2) student-student; (3) student-content*	(1), (3), (2)	(1), (2), (3)	(1), (2), (3)	(3), (1), (2)	(3), (1), (2)

### Participants

At the beginning of the course, students were asked to sign a consent form approved by the university’s Institutional Review Board. Of the students who agreed to participate in research (*n =* 326), the majority (85%) were STEM majors in programs offered through the institution’s primary colleges of science. Other majors represented were Arts (4.6%), Journalism and Mass Communication (2.1%), Education and Human Sciences (2.1%), Fine and Performing Arts (1.5%), Business Administration (1.2%), Agricultural Economics (1.2%), Architecture (0.9%), Public Affairs and Communication Services (0.3%), General studies (0.3%), and high school (0.6%). Over 2/3s of the students in the course were sophomore (30.1%) or juniors (36.8%), while 23.6% were seniors, and close to 10% were freshman (9.4%), post baccalaureates (0.3%) or high school students (0.6%) who joined the course as part of a state program. Students were relatively equal by gender, with 54% male, and 46% female (Appendix 1).

### Data sources

This study is based on the data from three course assessments, assignments, and instruments. First, the Pre-/Post-test is an assessment consisting of 41 questions administered at the beginning and end of the course to evaluate the students’ understanding of hydrology-related concepts. Second, the Water Balance Model (WBM) is a data-driven, computer-based modeling tool designed to support student’s understanding of factors affecting the hydric balance. Students’ experience with the WBM is through a multi-week project where they engaged with a scenario where they had to evaluate the allocation of irrigation acreage considering climatic zones, the water table, rainfall, runoff, and the potential evapotranspiration. Lally & Forbes, 2019 developed a scoring rubric which evaluates students’ use and evaluation of the model. In the study, we used the score for model use which evaluates students’ use of the model to introduce evidence, provide a detailed and complex description and explanation, generalize, predict or hypothesize, organize their ideas, and generate new information in their analysis about the phenomenon. Third, the Mid-Semester Evaluation (MSE) is a course evaluation survey involving Likert scale and open-ended questions, administered at the mid-point of the semester. Except for the MSE, data is available for all five years. Each of these instruments are described more fully in prior publications (Forbes et al., 2018; Lally & Forbes 2019, 2020). The number of students who responded to the instruments varied for each year and instrument (Table 2).

**Table 2:** Number of students who responded to the assessments

	2017	2018	2019	2020	2021
Number of students who participated in the study	45	61	58	48	114
Data sources					
Pre-test	45	55	46	44	114
Post-test	45	55	46	47	114
Water Balance Model	38	56	47	46	108
Mid-Semester Evaluation	41	58	-	46	111

### Data analysis

We calculated gain scores between the Pre and Post-tests to assess the differences between them. Then, we obtained descriptive statistics of all the quantitative scores using R program version 3.6.2. We performed ANOVAs to evaluate changes over time for the gain scores, Water Balance Model use scores, and the Mid-Semester Evaluation. In the cases in which we found statistically significant differences, we applied Tukey HSD Tests to identify the differences between years using R. Given the number of pairwise comparisons, we used the Bonferroni correction to establish the threshold for significance at *p* = .008 for post-hoc tests within each ANOVA.

To identify factors that would predict the gains or losses in students’ post-test scores, we performed an Analysis of Covariance (ANCOVA) where we included the categorical factors of gender, academic level and Major (STEM or Non-STEM), and all two-way interactions, and the covariate is the students’ pre-test scores. Pre-test scores have a negative linear relationship with gain scores setting a cap to students’ gain scores, where students who started the course with higher levels of knowledge about water concepts would be able to experience modest gains, whereas students with lower pre-test scores would be able to experience higher gains. In the cases in which we found statistically significant differences, we applied Tukey HSD Tests to identify the differences between the categorical variables. Furthermore, for the variables where we found differences, we ran ANOVAS to compare differences between years. These analyses were done using R program version 3.6.2. The full set of descriptive statistics, ANOVAS and corresponding Tukey HSD tests, and ANCOVA and corresponding Tukey HSD test are summarized in Appendix 2; 3, 4, 5, 6, 7, 8; and 9, respectively.

To analyze the qualitative data from the MSE, we evaluated, coded, and categorized each response to perform a Strengths, Weaknesses, Opportunities, and Threats (SWOT) analysis about students’ perceptions of the course. The SWOT analysis complemented the quantitative analysis from the MSE. First, we organized students’ comments by year, academic level, and Major using Word. Each comment received a number based on the order in which it was reviewed. We developed a code sheet using Excel with “strengths”, “weaknesses”, “opportunities”, and “threats” as main categories. We added columns representing the year, academic level, and Major to help keep track each response. As we read each response, we identified emergent sub-categories using representative words and assigned each comments’ number to a corresponding category and sub-category. When a comment represented two or more categories or sub-categories, it was added to the corresponding sections. As we read each comment, we also highlighted those that provided an ample description of the student’s opinions on the topic being discussed. With support of the code sheet, we developed a summary of strengths, weaknesses, opportunities, and threats for each question and year and re-organized the students’ responses accordingly using Word. The summary helped us identify students’ steady and changing perceptions of the course for each year. While developing the narrative, we traced back to the students’ responses and selected from those that were highlighted the most representative ones.

## Results

### Students’ understanding of hydrologic concepts

**Fig. 1:**
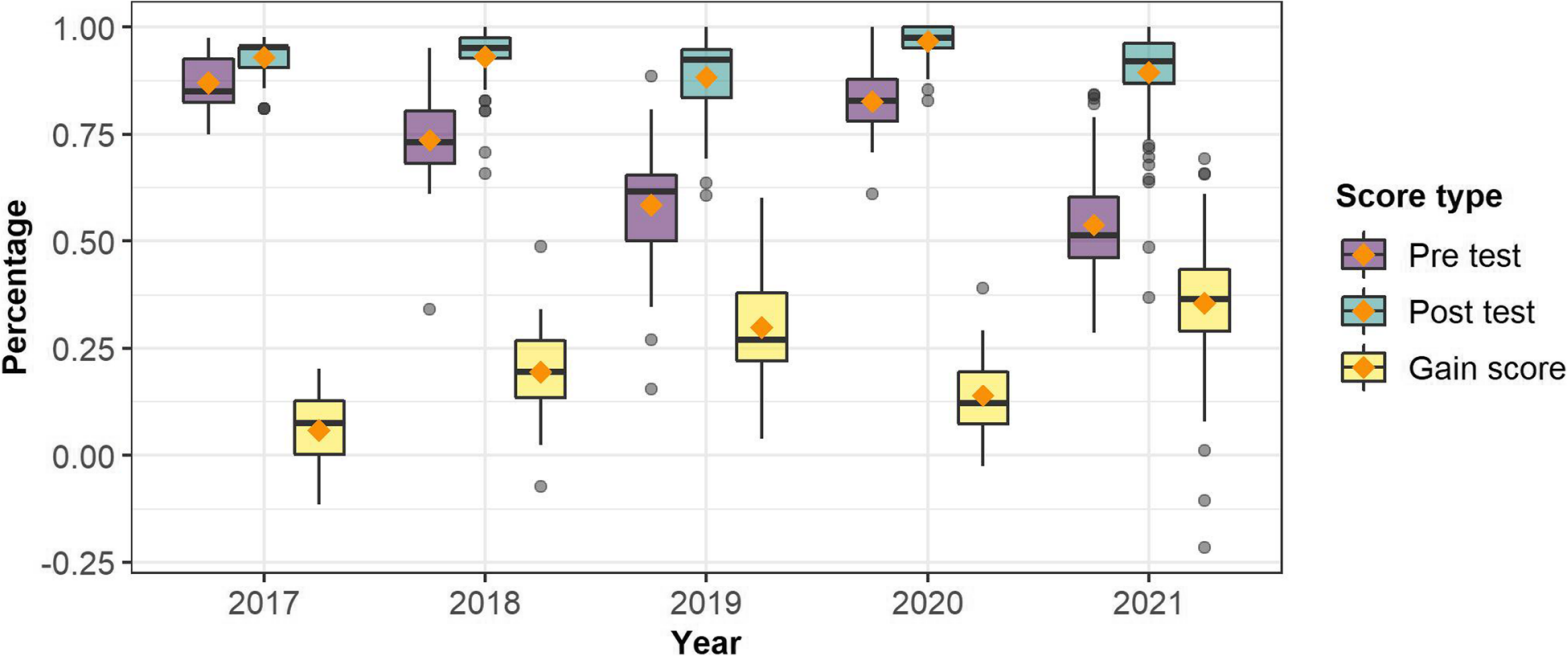
Pre-test, Post-test, and Gain scores (shown as percentages)

Analysis of students’ Pre-tests, Post-tests, and gain scores show students’ Pre-test and gain scores varied across years while their Post-test scores were relatively consistent (Fig. 1). For the Pre-test, the Post-test, and the gain scores, the effect of year was statistically significant (Table 3). We observed that across all five years, students began the course each year with varying levels of knowledge of hydrologic concepts. Students’ knowledge of core hydrological concepts at the beginning of the semester was highest and equivalent in 2017 and 2020; while it was relatively lower in 2018, and particularly, 2019 and 2021. The 2019 group and 2021 had the lowest average score and the highest standard deviation between students whereas the 2017 and 2020 groups had the highest average scores and lowest standard deviations (Fig. 1 & Table 4). Differences between 2017 and 2020, and 2019 and 2021 Pre-test scores were not statistically significant at *p* = .008. However, all other pairwise comparisons across year were statistically significant at *p* = .008.

**Table 3:** Pre-test, post-test, and change scores’ ANOVAs

Test scores	DFn	DFd	F	p
Pre-test scores	4	299	116.00	0.000
Post-test scores	4	302	8.83	0.000
Gain scores	4	298	65.37	0.000

**Table 4:** Pre-test scores descriptive statistics

Year	*n*	*M*	*SD*	*Min*	*Max*
2017	45	0.87	0.06	0.75	0.98
2018	55	0.74	0.10	0.34	0.95
2019	46	0.58	0.14	0.15	0.88
2020	47	0.83	0.08	0.61	1.00
2021	114	0.54	0.12	0.29	0.84

Compared to Pre-test scores, students’ scores on the Post-test were higher, more consistent, and relatively more similar to one another. By the end of the course, the students in the 2020, 2018 and 2017 had the least heterogeneity among students. The differences between the 2018 and 2019; 2019 and 2020; and 2020 and 2021 pairwise comparisons were statistically significant at *p* = .008. The 2019 and the 2021 groups exhibited the lowest Post-test scores (Table 5). These findings suggest that students in each year left the course with relatively similar levels of understanding of core hydrological concepts, except for 2019 and 2021 which exhibited Post-test scores that were approximately 5% lower than 2018, and 9% and 7% lower than 2020, respectively.

**Table 5:** Post-test scores descriptive statistics

Year	*n*	*M*	*SD*	*Min*	*Max*
2017	45	0.93	0.04	0.81	0.98
2018	55	0.93	0.07	0.66	1.00
2019	46	0.88	0.10	0.61	1.00
2020	47	0.97	0.04	0.83	1.00
2021	114	0.90	0.11	0.37	1.00

Analysis of the gain scores show that students’ knowledge of core hydrological concepts increased each year; however, the magnitude of these increases was different every year. We observed that between all possible pairs of years, except for 2018 and 2020, the mean differences were statistically-significant at *p* = .008. The 2021 (*M* = 0.36, *SD* = 0.14) and 2019 groups (*M* = 0.30, *SD* = 0.13) had the largest increase, but students’ individual gains were the most heterogeneous of all groups. It was followed by the 2018 group (*M* = 0.19, *SD* = 0.10) and 2020 group (*M* = 0.14, *SD* = 0.10) which were relatively similar. Gain scores for students in 2017 (*M* = 0.06, *SD* = 0.08) were the lowest (Fig. 1 & Table 6).

**Table 6:** Gain-scores descriptive statistics

Year	n	*M*	*SD*	*Min*	*Max*
2017	45	0.06	0.08	-0.12	0.20
2018	55	0.19	0.10	-0.07	0.49
2019	46	0.30	0.13	0.04	0.60
2020	43	0.14	0.10	-0.02	0.39
2021	114	0.36	0.14	-0.22	0.69

Finally, results from the ANCOVA show that the effect of the Major (STEM or Non STEM) on gain scores was statistically significant at *p <* .01; whereas the effects of academic level, gender, and the interactions between Major and academic level were statistically significant at *p* < .05 (Table 7). The Tukey HSD tests showed differences in Major, but not in the other variables at *p* < .05. The ANOVA results show that the effect of year for the Major was statistically significant at *p <* .05 (Table 7). In 2018, STEM students (*M* = 0.21, *SD* = 0.09) obtained larger gains than Non-STEM students (*M* = 0.12, *SD* = 0.13) in the same cohort. To explain these differences, in 2018, Non-STEM students already had a high pre-test scores (*M* = 0.79, *SD* = 0.10) compared to STEM students (*M* = 0.73, *SD* = 0.10), thus limiting their gain scores. The post-test scores for Non-STEM students (*M* = 0.91, *SD* = 0.11) suggest that they obtained modest improvements, and these were close to STEM students (*M* = 0.93, *SD* = 0.06). To complement, in 2018, 7 out of 8 of Non-STEM students (87.5%) were Juniors or Seniors compared to a more diverse group of STEM students (60% were from Post-baccalaureate to Sophomore, and 40% were Juniors or Seniors). Results from the qualitative analysis indicate that their learning might have been already covered in a combination of previous courses in the past. As a Junior student indicated, *“I think the activities are important, but they have been covered in other courses for me”.* No other significant differences were observed. These findings suggest that students, regardless of their major, academic level, and gender, were able to improve their initial knowledge of hydrologic concepts.

**Table 7:** ANCOVA* and ANOVA** results

Effect	DFn	DFd	F	p
Major*	1	283	7.40	.007
Academic level*	3	283	3.81	.011
Gender*	1	283	6.65	.010
Major: Academic level*	3	283	3.08	.028
Major**	1	52	4.16	.046

### Modeling assignment

**Fig. 2:**
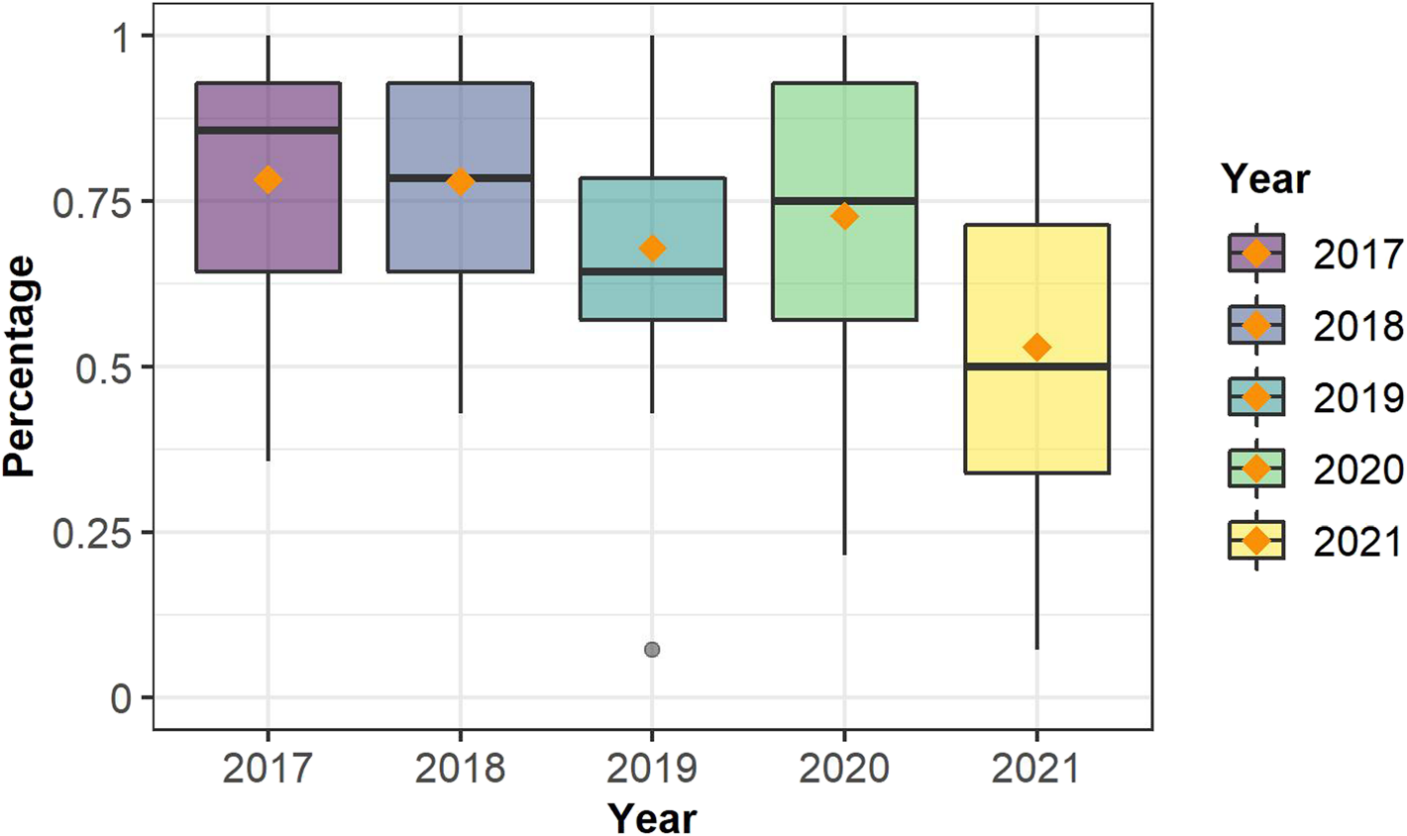
Water Balance Model’s use scores (shown as percentages)

Analysis of the modeling assignment show that students’ use scores for the Water Balance Model (WBM) between 2017, 2018, 2019 , and 2020 were relatively similar, as shown in Fig. 2 and Table 8. However, the 2021 group, *F* (4,290) = 18.43, *p* = 0.00, exhibited statistically significant differences with the previous years at *p=*.008. Students in this group scored the lowest compared to other years and showed the largest heterogeneity among groups, with the first and third quartile ranging from 0.34 to 0.71 on a percentual scale. No other statistically significant differences were observed across years for the modeling assignment. These results suggest that while students’ model use in 2021 was the lowest, students’ model-based use was relatively similar across previous years.

**Table 8:** Water Balance Model descriptive statistics

Year	*n*	*M*	*SD*	*Min*	*Max*
2017	38	0.78	0.19	0.36	1.00
2018	56	0.78	0.18	0.43	1.00
2019	47	0.68	0.20	0.07	1.00
2020	46	0.73	0.23	0.21	1.00
2021	108	0.53	0.24	0.07	1.00

### Students’ overall perceptions of the course

**Fig. 3:**
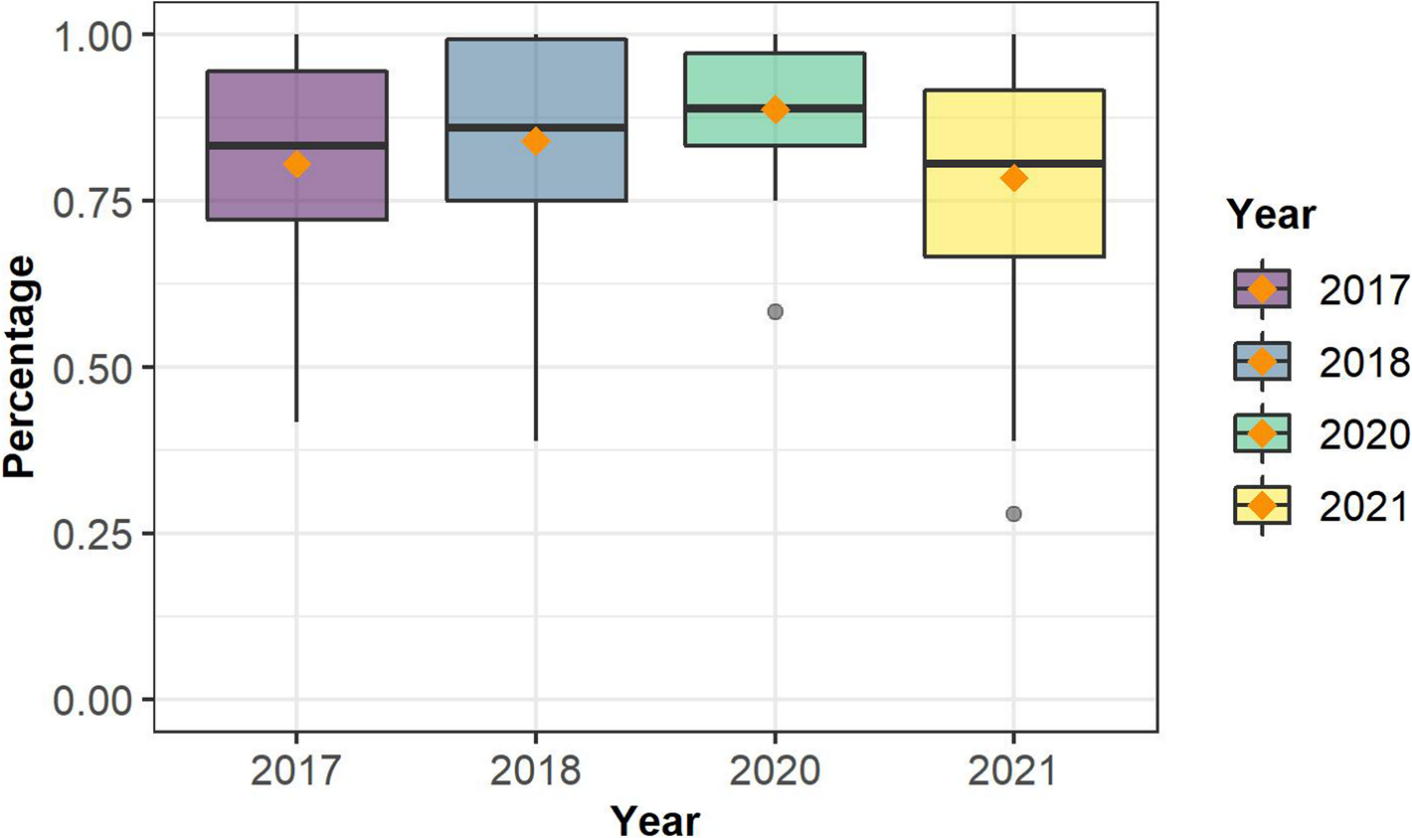
Mid Semester Total Evaluation scores (shown as percentages)

In general, the analysis of the mid-semester course evaluation showed that the effect of year on student evaluation score was statistically significant, *F* (3,252) = 5.73, *p* = .000. The mean differences between 2020 and 2021 were statistically significant at *p* = .008. No other statistically significant differences were found. In this sense, it is possible to observe that students’ overall satisfaction with the course increased from 2017 to 2020. Considering a percentual scale, students evaluated the course the highest and most homogeneously in 2020, followed by 2018, and 2017. However, students’ evaluation dropped off in 2021 compared to 2020 (Fig. 3 & Table 9).

**Table 9:** Mid Semester Total Evaluation scores’ descriptive statistics

Year	*n*	*M*	*SD*	*Min*	*Max*
2017	41	0.81	0.15	0.42	1.00
2018	58	0.84	0.15	0.39	1.00
2020	46	0.89	0.09	0.58	1.00
2021	111	0.79	0.16	0.28	1.00

Results from the MSE suggest that over the years, students’ overall perception of the course was highly positive. As the course evolved, students' perceptions about the level of learning and opportunities to learn and provide different viewpoints improved all years (students’ agreement increased from 87% in 2017 to 93% in 2021, and from 85% in 2017 to 94% in 2021, respectively), and seem to have been the least affected by the transitions brought by COVID-19, as in 2021 students’ perception was less than 1% lower than the previous year. In this sense, the course has maintained an overarching value related to the importance of water for everyday life. In 2017 a student commented *“I have learned a lot about how water contributes to more than just keeping humans hydrated, which is a good thing because I did not know how reliant we are on such a natural resource!”.* The course also provided multiple views about water that helped students construct their own opinions and evaluate critically their own decisions. With regards to improvements, between 2017 to 2020, some students noted that the course could benefit from more in-depth content, especially regarding the social components of water, and the inclusion of cases addressing countries outside of the United States. In 2017, a student commented *“I was expecting to go in depth on water policy and environmental justice issues throughout this course...”.* Another student in 2017, who as an international student, commented that *“it’s just hard to know where I would apply [the activities from the course] in my country”*. In response, contents of the course were improved, and by 2021, students’ comments reflected how they valued the depth and breadth of water-related topics delivered through the videos and readings, and the introduction of global, national, and local cases. In that year, a student commented. *“I am learning about water issues on the global level which I think is new to me”.*

Other factors that seem to have been least affected by COVID-19 are the usefulness of the course to validate students’ current interests and even guide future career (students’ agreement increased from 75% in 2007 to 87% in 2021); the importance of course assessments as an important learning tool to review, clarify and demonstrate students’ learnings with a proper level of difficulty, application of decision-making skills to real-cases, and metacognition (students’ agreement increased from 78% in 2017 to 92% in 2021); and the learning outcomes (students’ agreement increased from 79% in 2017 to 93% in 2021) also improved over the years. These aspects showed a smaller than 2% difference in students’ agreement in 2021 compared to 2020. However, in 2020 and 2021, with the transition to a fully online and asynchronous mode, students experienced challenges with the assessments of the course. They expressed that, even with a similar workload compared to previous years, the daily assignments could be grouped together to reduce the workload and cognitive load. In 2020, a student said *“... If I were to change something about the course, I would start with the duration of the assignments or the amount of work the students do”.* Other improvements in the assessments identified by the students over the years include the need for more thorough explanations of the instructions, and time to develop them.

Students evaluated the team of instructors and learning assistants positively every year (students agreement increased from 89% in 2017 to 94% in 2020), and highlighted their expertise and pedagogy, support and guidance, engagement and delivery, feedback, and openness to address different opinions. However, these are areas where the impacts of COVID-19 are more visible in 2021, as there was a reduction in 4% to 5% of students’ perceptions compared to 2020. In 2021, as a result of the online and asynchronous mode, there was less teacher-student interaction, and less real-time opportunities to provide clarification which may have impacted students’ overall interaction with the course. In 2021 a student commented *“This might be because of difficulties adjusting to COVID-19, but the online course is hard to be engaged and interested in”.* Still, students valued their support and guidance through the feedback on assignments, and a Question-and-Answer module made accessible on Canvas to respond to students’ inquiries. With a higher degree of student-content interaction, the course videos were also important to support students’ engagement. In this sense, students recommended providing more emphasis to communication between instructors and students. Also, providing additional supports through a variety of course materials can help address some of the challenges of an asynchronous mode. As a student in 2021 indicated *“I like how this class brings different types of material -- readings, videos, websites, etc. to students. This facilitates the assimilation and understanding of the key concepts being taught as the students view the learning process as dynamic”.*

Working in a group environment was the area that seems most affected by the course transition resulting from COVID-19. Between 2017 and 2020 students’ perceptions about working in a group environment improved (students’ agreement increased from 80% in 2017 to 96% in 2020). However, there was a drop in 2021 compared to 2020 of 31%. In 2021, students emphasized the challenges reaching out to other students and coordinating group meetings online due to lack of responsiveness of other group members. A student shared *“Group learning can be incredibly difficult in an online setting. I was lucky and got a really great group, but still online group work can be hard to organize from the student perspective”.* Other challenges that were also reflected in previous years include the lack of cooperation and reliability of some group members; and the reduced opportunities for discussing ideas. A student in 2021 commented *“... doing the infographic 100% asynchronously really allowed people to slack off. It was very difficult to maintain communication”.* Still, over the years, including 2021, students highlighted that working in a functional group environment facilitated learning. Another affordance of the group environment is that it also enables students to learn and exchange different perspectives from various backgrounds. Students also highlighted that the group environment helped them develop personal and teamwork skills. Recommendations to support the group environment, particularly in an online setting, include providing more guidance, and follow-up to students. As a student in 2021 commented *“[W] hen group projects are given the [re] should be more guidance given to allow for a better team-work”*.

## Discussion

Water education is important to support students to develop water literacy, including knowledge, skills, attitudes, and behaviors to developing sustainable water management practices (Attari et al., 2017; King et al., 2012; McCarroll & Hamann, 2020; Su et al., 2011). This study builds upon prior research in the context of the Water in Society course (Forbes et al., 2018; Lally et al., 2020; Lally & Forbes, 2019, 2020; Owens et al., 2020; White & Forbes, 2021), our broader undergraduate water education efforts (Petitt & Forbes, 2019; Sabel et al., 2017), as well as research in the field (AghaKouchak et al., 2013; Arthurs & Elwonger, 2018; Attari et al., 2017; Cardak, 2009; Covitt et al., 2009; Gunn et al., 2002; Habib et al., 2012; Johnson & Courter, 2020; King et al., 2012; Kingston et al., 2012; Li & Liu, 2003; Li et al., 2016; Merwade & Ruddell, 2012; Sibley et al., 2007; Smith et al., 2006; Su et al., 2011; Wagener et al., 2012; White et al., 2017; Willermet et al., 2013; Williams et al., 2009; Zigic & Lemckert, 2007). It provides important insight into efforts to cultivate undergraduate students’ water literacy through different course formats and the potential impacts of transitioning to a more student-content interaction (Li et al., 2016) as a result of the COVID-19 pandemic.

First, research has shown that students and adults may not hold scientifically accurate conceptions about water (Arthurs & Elwonger, 2018; Attari et al., 2017; Cardak, 2009; Covitt et al., 2009; Duda et al., 2005; Johnson & Courter, 2020; Sibley et al., 2007; Williams et al., 2009). Based on the results from the analysis of the Pre-/Post-tests, it is encouraging to see that each year the course has been effective in helping students develop introductory disciplinary knowledge, regardless of their initial understanding of hydrologic concepts, academic level, major, and gender, even with the transition to a fully online environment. Furthermore, the course has exhibited not only a high level of consistency in helping students attain what we believe a reasonable (88%) to high level (93% and above) of disciplinary understanding at the introductory level, but also engagement in data-driven modeling of real-world, water-related scenarios through the modeling assignment, for which students’ performance was similar across Years 1-4. While the relative consistency of these student outcomes can be interpreted as a positive, and we did observe some modest improvements in modeling sub-processes in a previous study (Lally & Forbes, 2019), it also suggests that changes to the course over time, particularly its increasingly hybrid nature and student-centeredness, did not substantially enhance student’s overall model-based reasoning as intended, at least as measured through the WBM task. We hypothesize that independent of the course format and structure, students may need various kinds of support learning to use and interpret the results from data-driven, computer-based models more effectively. Future research should continue to investigate what these supports are and how they can be employed to enhance undergraduate students’ learning about water across course formats.

Second, study findings show that students’ satisfaction with the course increased between 2017 to 2020. We interpret this result as affirmation of the more student-centered, research-based, and flipped nature of the course as it evolved over the five years. Prior research has shown that students respond favorably to research-based practices in undergraduate classrooms, including the use of class meetings for active learning, engagement with instructors, peers, and learning assistants, and collaborative problem-solving, all of which contrast with more traditional, one-directional, lecture-based classroom climates (Habib et al., 2012; Halvorson & Westcoat, 2002; Kingston et al., 2012; Li & Liu, 2003; Smith et al., 2006; Thompson et al., 2012; White et al., 2017; Willermet et al., 2013). In an engaging and learner-centric classroom, students are better positioned to internalize the importance of water resources management for their personal and professional future endeavors, recognize the relevance of the course to guide them in their selection of a water-related career, to develop decision-making skills, and to work with interdisciplinary teams. In general, this finding supports the idea that students generally respond favorably to student-centered undergraduate learning environments that foreground active learning and student engagement.

Third, study findings also yield insights into the impacts of the COVID-19 pandemic on undergraduate STEM education. As shown in the results, students’ satisfaction with the course decreased in 2021 after consistently increasing each year between 2017 to 2020. Additionally, in 2021, we observed a sizeable decrease in students’ performance on the WBM assignment, a critical opportunity for students to learn to analyze SHIs, after relatively stable outcomes across Years 1-4 (AghaKouchak et al., 2013; Attari et al., 2017; Handelsman et al., 2004; Gunn et al., 2002; King et al., 2012; Lally & Forbes, 2019, 2020; Merwade & Ruddell, 2012; Sibley et al., 2007; Zigic & Lemckert, 2007). We believe these results to be attributable to disruptions brought about by COVID-19 for several specific reasons. Changes to the course in Year 5 (2021) fundamentally altered dynamics of collaboration between instructors and students, including peer interactions, especially as compared to the increasingly flipped and student-centered course model in Years 2-5. The decreases in students’ model-based reasoning and perceptions of the course in 2021 might have resulted from the transition to more participant-content interaction in which students mostly engaged with course materials and assignments and had less engagement with the instructor, teaching assistants, and peers in an asynchronous environment (Li et al., 2016). Although different opportunities for communication were provided, students may need additional encouragement to take advantage of these resources. Communication among peers is another area that may require additional support in an online and asynchronous setting. We also recognize the increasing levels of pandemic fatigue from which students were suffering in spring, 2021, by which time students had been experiencing a year or more of pandemic-related disruptions. The rapid and often uneven transition to a mostly or fully virtual undergraduate experience brought about by the pandemic was jarring and presented challenges for instructors and students alike. As such, findings from this research suggest that conditions brought about by COVID-19 may have impacted students’ experience with the course as compared to previous years.

### Limitations and future research

While the present study illustrates longitudinal outcomes in undergraduate STEM education, including potential impacts of the COVID-19 pandemic, it is limited in a number of ways. First, it highlights only a subset of potential water-related student learning outcomes we may seek to cultivate. Subsequent research should explore additional outcomes related to undergraduate students’ learning about water systems and SHIs. Second, it is limited in scope to the specific population of students served by the course. Future research could replicate and elaborate upon the present study by focusing on larger and different student populations, which may provide insight into learning needs of particular groups of students. Such research may involve inclusion of additional student-level variables, such as access to technology, household socio-economic status, and affective orientations towards STEM, as well as course-related variables, such as communication practices with instructors, experience working in more independent environments, and engagement with other course elements, all of which may have contributed to challenges students experienced during the course amid the global pandemic. This work would help clarify how the course’s characteristics may have afforded and/or constrained students’ learning in the midst of COVID-19 and identify additional curricular and instructional supports for students.

## Conclusion

Water-related issues remain one of the greatest challenges facing the global community. Education, and particularly undergraduate STEM education, has a critical role to play in adequately preparing individuals to problem-solve and address SHIs in varying domains of everyday life. Results presented here showcase one effort to prepare students to reason about real-world issues and how these efforts were impacted by another global challenge: the COVID-19 pandemic. As such, this research provides important contributions to the design and delivery of innovative undergraduate STEM educational interventions, including those foregrounding disciplinary concepts and model-based reasoning about socio-hydrologic systems, under unique and challenging educational circumstances brought about by the COVID-19 pandemic.

## Supplementary Information

**Additional file 1: Appendix 1.** Students’ demographics.

**Additional file 2: Appendix 2.** Descriptive statistics.

**Additional file 3: Appendix 3.** Pre-test scores: (a) ANOVAs and (b) Tukey HSD tests.

**Additional file 4: Appendix 4.** Post-test scores: (a) ANOVAs and (b) Tukey HSD tests.

**Additional file 5: Appendix 5.** Gain scores: (a) ANOVAs and (b) Tukey HSD tests.

**Additional file 6: Appendix 6.** Water Balance Model: (a) ANOVAs and (b) Tukey HSD tests.

**Additional file 7: Appendix 7.** Mid-Semester Evaluation: (a) ANOVAs and (b) Tukey HSD tests.

**Additional file 8: Appendix 8.** Major: ANOVA.

**Additional file 9: Appendix 9.** (a) ANCOVA: Type III Tests of Fixed effects and (b) Tukey HSD test.

## Data Availability

The datasets used and/or analyzed during the current study are available from the corresponding author on reasonable request.
