# Supplementary material for: Supporting undergraduate students’ developing water literacy during a global pandemic: a longitudinal study
Source: Discip Interdscip Sci Educ Res. 2022 Mar 7;4(1):7. doi: 10.1186/s43031-022-00049-y (PMC8899452; doi:10.1186/s43031-022-00049-y)
Supplement: Supplementary file 1 — Additional file 1: Appendix 1. Students’ demographics. [file 43031_2022_49_MOESM1_ESM.docx]

Appendix 1.

*Students’ demographics*

|  | 2017 | 2018 | 2019 | 2020 | 2021 | Total |
| --- | --- | --- | --- | --- | --- | --- |
| Students’ Program |  |  |  |  |  |  |
| Agricultural and Natural Resources | 35 | 53 | 47 | 44 | 68 | 247 |
| Arts and Sciences* | 5 | 5 | 7 | 2 | 22 | 41 |
| Architecture | - | - | - | - | 3 | 3 |
| Business Administration | - | - | 1 | 1 | 2 | 4 |
| Education and Human Sciences | 3 | 1 | - | - | 3 | 7 |
| Engineering | - | - | 1 | 1 | 6 | 8 |
| Fine and Performing Arts | - | - | 1 | - | 4 | 5 |
| General Studies | 1 | - | - | - | - | 1 |
| Journalism and Mass Communication | 1 | 2 | - | - | 4 | 7 |
| Public Affairs and Communication Services | - | - | 1 | - | - | 1 |
| Undefined | - | - | - | - | 2 | 2 |
| *Total* | *45* | *61* | *58* | *48* | *114* | *326* |
| Students’ Academic level |  |  |  |  |  |  |
| Freshman | 11 | 2 | 5 | 2 | 8 | 28 |
| Sophomore | 15 | 30 | 21 | 4 | 28 | 98 |
| Junior | 11 | 16 | 20 | 28 | 45 | 120 |
| Senior | 8 | 12 | 12 | 14 | 31 | 77 |
| Post-baccalaureate | - | 1 | - | - | - | 1 |
| High School Student | - | - | - | - | 2 | 2 |
| Students’ Gender |  |  |  |  |  |  |
| Female | 19 | 35 | 24 | 24 | 47 | 149 |
| Male | 26 | 26 | 34 | 24 | 67 | 177 |

Note: *In the methods, students in Arts and Sciences were re-classified based on their Career/Program/Plan characteristics. 12 students were included in the STEM group, 6 in Education and Human Sciences, and 1 in Public Affairs and Communication Services.
