# Supplementary material for: Supporting undergraduate students’ developing water literacy during a global pandemic: a longitudinal study
Source: Discip Interdscip Sci Educ Res. 2022 Mar 7;4(1):7. doi: 10.1186/s43031-022-00049-y (PMC8899452; doi:10.1186/s43031-022-00049-y)
Supplement: Supplementary file 2 — Additional file 2: Appendix 2. Descriptive statistics. [file 43031_2022_49_MOESM2_ESM.docx]

Appendix 2.

*Descriptive statistics*

| Assessment | Year | *n* | *M* | *SD* | *Min* | *Max* |
| --- | --- | --- | --- | --- | --- | --- |
| Pre-test | 2017 | 45 | 0.87 | 0.06 | 0.75 | 0.98 |
|  | 2018 | 55 | 0.74 | 0.10 | 0.34 | 0.95 |
|  | 2019 | 46 | 0.58 | 0.14 | 0.15 | 0.88 |
|  | 2020 | 47 | 0.83 | 0.08 | 0.61 | 1.00 |
|  | 2021 | 114 | 0.54 | 0.12 | 0.29 | 0.84 |
| Post-test | 2017 | 45 | 0.93 | 0.04 | 0.81 | 0.98 |
|  | 2018 | 55 | 0.93 | 0.07 | 0.66 | 1.00 |
|  | 2019 | 46 | 0.88 | 0.10 | 0.61 | 1.00 |
|  | 2020 | 47 | 0.97 | 0.04 | 0.83 | 1.00 |
|  | 2021 | 114 | 0.90 | 0.11 | 0.37 | 1.00 |
| Gain scores | 2017 | 45 | 0.06 | 0.08 | -0.12 | 0.20 |
|  | 2018 | 55 | 0.19 | 0.10 | -0.07 | 0.49 |
|  | 2019 | 46 | 0.30 | 0.13 | 0.04 | 0.60 |
|  | 2020 | 43 | 0.14 | 0.10 | -0.02 | 0.39 |
|  | 2021 | 114 | 0.36 | 0.14 | -0.22 | 0.69 |
| Water Balance Model | 2017 | 38 | 0.78 | 0.19 | 0.36 | 1.00 |
|  | 2018 | 56 | 0.78 | 0.18 | 0.43 | 1.00 |
|  | 2019 | 47 | 0.68 | 0.20 | 0.07 | 1.00 |
|  | 2020 | 46 | 0.73 | 0.23 | 0.21 | 1.00 |
|  | 2021 | 108 | 0.53 | 0.24 | 0.07 | 1.00 |
| Mid semester evaluation | 2017 | 41 | 0.81 | 0.15 | 0.42 | 1.00 |
|  | 2018 | 58 | 0.84 | 0.15 | 0.39 | 1.00 |
|  | 2020 | 46 | 0.89 | 0.09 | 0.58 | 1.00 |
|  | 2021 | 111 | 0.79 | 0.16 | 0.28 | 1.00 |
| Gain scores for non-STEM track students | 2017 | 8 | 0.03 | 0.05 | -0.05 | 0.10 |
|  | 2018 | 6 | 0.11 | 0.13 | -0.07 | 0.27 |
|  | 2019 | 5 | 0.35 | 0.10 | 0.27 | 0.50 |
|  | 2020 | 2 | 0.14 | 0.09 | 0.07 | 0.20 |
|  | 2021 | 24 | 0.34 | 0.12 | 0.01 | 0.57 |
| Gain scores for STEM track students | 2017 | 37 | 0.07 | 0.09 | -0.12 | 0.20 |
|  | 2018 | 54 | 0.20 | 0.09 | 0.02 | 0.49 |
|  | 2019 | 42 | 0.29 | 0.14 | 0.04 | 0.60 |
|  | 2020 | 46 | 0.14 | 0.10 | -0.02 | 0.39 |
|  | 2021 | 88 | 0.36 | 0.15 | -0.21 | 0.69 |
