# Supplementary material for: Supporting undergraduate students’ developing water literacy during a global pandemic: a longitudinal study
Source: Discip Interdscip Sci Educ Res. 2022 Mar 7;4(1):7. doi: 10.1186/s43031-022-00049-y (PMC8899452; doi:10.1186/s43031-022-00049-y)
Supplement: Supplementary file 3 — Additional file 3: Appendix 3. Pre-test scores: (a) ANOVAs and (b) Tukey HSD tests. [file 43031_2022_49_MOESM3_ESM.docx]

Appendix 3.

*Pre-test scores: (a) ANOVAs and (b) Tukey HSD tests*

| (a) | Effect | DFn | DFd | F | p | p<.008 |
| --- | --- | --- | --- | --- | --- | --- |
|  | Year | 4 | 299 | 116.00 | 0.000 | * |
| (b) |  |  |  |  |  |  |
| Group1 | Group2 | Estimate | Conf.low | Conf.high | p.adj | p<.008 |
| 2017 | 2018 | -0.13 | -0.19 | -0.08 | 0.000 | * |
| 2017 | 2019 | -0.29 | -0.34 | -0.23 | 0.000 | * |
| 2017 | 2020 | -0.04 | -0.10 | 0.01 | 0.335 | ns |
| 2017 | 2021 | -0.33 | -0.38 | -0.28 | 0.000 | * |
| 2018 | 2019 | -0.15 | -0.20 | -0.10 | 0.000 | * |
| 2018 | 2020 | 0.09 | 0.04 | 0.14 | 0.000 | * |
| 2018 | 2021 | -0.20 | -0.25 | 0.15 | 0.000 | * |
| 2019 | 2020 | 0.24 | 0.19 | 0.30 | 0.000 | * |
| 2019 | 2021 | -0.05 | -0.10 | 0.01 | 0.110 | ns |
| 2020 | 2021 | -0.29 | -0.34 | -0.24 | 0.000 | * |
