# Supplementary material for: Supporting undergraduate students’ developing water literacy during a global pandemic: a longitudinal study
Source: Discip Interdscip Sci Educ Res. 2022 Mar 7;4(1):7. doi: 10.1186/s43031-022-00049-y (PMC8899452; doi:10.1186/s43031-022-00049-y)
Supplement: Supplementary file 4 — Additional file 4: Appendix 4. Post-test scores: (a) ANOVAs and (b) Tukey HSD tests. [file 43031_2022_49_MOESM4_ESM.docx]

Appendix 4.

*Post-test scores: (a) ANOVAs and (b) Tukey HSD tests*

| (a) | Effect | DFn | DFd | F | p | p<.008 |
| --- | --- | --- | --- | --- | --- | --- |
|  | Year | 4 | 302 | 8.83 | 0.000 | * |
| (b) |  |  |  |  |  |  |
| Group1 | Group2 | Estimate | Conf.low | Conf.high | p.adj | p<.008 |
| 2017 | 2018 | 0.00 | -0.04 | 0.05 | 1.000 | ns |
| 2017 | 2019 | -0.05 | -0.09 | 0.00 | 0.061 | ns |
| 2017 | 2020 | 0.04 | -0.01 | 0.08 | 0.206 | ns |
| 2017 | 2021 | -0.04 | -0.08 | 0.00 | 0.117 | ns |
| 2018 | 2019 | -0.05 | -0.09 | -0.00 | 0.035 | * |
| 2018 | 2020 | 0.04 | -0.01 | 0.08 | 0.195 | ns |
| 2018 | 2021 | -0.04 | 0.04 | 0.00 | 0.060 | ns |
| 2019 | 2020 | 0.08 | 0.03 | 0.13 | 0.000 | * |
| 2019 | 2021 | 0.01 | -0.03 | 0.05 | 0.937 | ns |
| 2020 | 2021 | -0.07 | -0.11 | -0.03 | 0.000 | * |
