# Supplementary material for: Supporting undergraduate students’ developing water literacy during a global pandemic: a longitudinal study
Source: Discip Interdscip Sci Educ Res. 2022 Mar 7;4(1):7. doi: 10.1186/s43031-022-00049-y (PMC8899452; doi:10.1186/s43031-022-00049-y)
Supplement: Supplementary file 5 — Additional file 5: Appendix 5. Gain scores: (a) ANOVAs and (b) Tukey HSD tests. [file 43031_2022_49_MOESM5_ESM.docx]

Appendix 5.

*Gain scores: (a) ANOVAs and (b) Tukey HSD tests*

| (a) | Effect | DFn | DFd | F | P | p<.008 |
| --- | --- | --- | --- | --- | --- | --- |
|  | Year | 4 | 298 | 65.37 | 0.000 | * |
| (b) |  |  |  |  |  |  |
| Group1 | Group2 | Estimate | Conf.low | Conf.high | p.adj | p<.008 |
| 2017 | 2018 | 0.13 | 0.06 | 0.20 | 0.000 | * |
| 2017 | 2019 | 0.24 | 0.17 | 0.31 | 0.000 | * |
| 2017 | 2020 | 0.08 | 0.01 | 0.15 | 0.002 | * |
| 2017 | 2021 | 0.30 | 0.24 | 0.35 | 0.000 | * |
| 2018 | 2019 | 0.10 | 0.04 | 0.17 | 0.000 | * |
| 2018 | 2020 | -0.06 | -0.12 | 0.01 | 0.153 | ns |
| 2018 | 2021 | 0.16 | 0.11 | 0.22 | 0.000 | * |
| 2019 | 2020 | -0.16 | -0.23 | -0.10 | 0.000 | * |
| 2019 | 2021 | 0.06 | 0.00 | 0.11 | 0.005 | * |
| 2020 | 2021 | 0.22 | 0.16 | 0.28 | 0.000 | * |
