# Supplementary material for: Supporting undergraduate students’ developing water literacy during a global pandemic: a longitudinal study
Source: Discip Interdscip Sci Educ Res. 2022 Mar 7;4(1):7. doi: 10.1186/s43031-022-00049-y (PMC8899452; doi:10.1186/s43031-022-00049-y)
Supplement: Supplementary file 6 — Additional file 6: Appendix 6. Water Balance Model: (a) ANOVAs and (b) Tukey HSD tests. [file 43031_2022_49_MOESM6_ESM.docx]

Appendix 6.

*Water Balance Model: (a) ANOVAs and (b) Tukey HSD tests*

| (a) | Effect | DFn | DFd | F | p | p<.008 |
| --- | --- | --- | --- | --- | --- | --- |
|  | Year | 4 | 290 | 18.43 | 0.000 | * |
| (b) |  |  |  |  |  |  |
| Group1 | Group2 | Estimate | Conf.low | Conf.high | p.adj | p<.008 |
| 2017 | 2018 | -0.09 | -3.58 | 3.40 | 1.000 | ns |
| 2017 | 2019 | -2.93 | -6.55 | 0.69 | 0.175 | ns |
| 2017 | 2020 | -1.56 | -5.19 | 2.08 | 0.766 | ns |
| 2017 | 2021 | -7.10 | -10.20 | -3.97 | 0.000 | * |
| 2018 | 2019 | -2.84 | -6.12 | 0.45 | 0.126 | ns |
| 2018 | 2020 | -1.47 | -4.77 | 1.84 | 0.740 | ns |
| 2018 | 2021 | -7.01 | -9.74 | -4.27 | 0.000 | * |
| 2019 | 2020 | 1.37 | -2.07 | 4.81 | 0.810 | ns |
| 2019 | 2021 | -4.17 | -7.07 | -1.27 | 0.000 | * |
| 2020 | 2021 | -5.54 | -8.46 | -2.62 | 0.000 | * |
