# Supplementary material for: Supporting undergraduate students’ developing water literacy during a global pandemic: a longitudinal study
Source: Discip Interdscip Sci Educ Res. 2022 Mar 7;4(1):7. doi: 10.1186/s43031-022-00049-y (PMC8899452; doi:10.1186/s43031-022-00049-y)
Supplement: Supplementary file 7 — Additional file 7: Appendix 7. Mid-Semester Evaluation: (a) ANOVAs and (b) Tukey HSD tests. [file 43031_2022_49_MOESM7_ESM.docx]

Appendix 7.

*Mid-Semester Evaluation: (a) ANOVAs and (b) Tukey HSD tests*

| (a) | Effect | DFn | DFd | F | P | p<.008 |
| --- | --- | --- | --- | --- | --- | --- |
|  | Year | 3 | 252 | 5.73 | 0.001 | * |
| (b) |  |  |  |  |  |  |
| Group1 | Group2 | Estimate | Conf.low | Conf.high | p.adj | p<.05 |
| 2017 | 2018 | 0.03 | -0.04 | 0.11 | 0.682 | ns |
| 2017 | 2020 | 0.08 | -0.00 | 0.16 | 0.053 | ns |
| 2017 | 2021 | -0.02 | -0.09 | 0.05 | 0.863 | ns |
| 2018 | 2020 | 0.05 | -0.03 | 0.12 | 0.360 | ns |
| 2018 | 2021 | -0.05 | -0.12 | 0.01 | 0.105 | ns |
| 2020 | 2021 | -0.10 | -0.17 | -0.04 | 0.001 | * |
