# Supplementary material for: Supporting undergraduate students’ developing water literacy during a global pandemic: a longitudinal study
Source: Discip Interdscip Sci Educ Res. 2022 Mar 7;4(1):7. doi: 10.1186/s43031-022-00049-y (PMC8899452; doi:10.1186/s43031-022-00049-y)
Supplement: Supplementary file 8 — Additional file 8: Appendix 8. Major: ANOVA. [file 43031_2022_49_MOESM8_ESM.docx]

Appendix 8.

*Major: ANOVA*

| Year | Effect | DFn | DFd | F | p | p<.05 |
| --- | --- | --- | --- | --- | --- | --- |
| 2017 | Major | 1 | 43 | 1.38 | 0.247 | ns |
| 2018 | Major | 1 | 52 | 4.16 | 0.046 | * |
| 2019 | Major | 1 | 44 | 0.93 | 0.340 | ns |
| 2020 | Major | 1 | 41 | 0.00 | 0.955 | ns |
| 2021 | Major | 1 | 110 | 0.38 | 0.537 | ns |
