# Supplementary material for: Supporting undergraduate students’ developing water literacy during a global pandemic: a longitudinal study
Source: Discip Interdscip Sci Educ Res. 2022 Mar 7;4(1):7. doi: 10.1186/s43031-022-00049-y (PMC8899452; doi:10.1186/s43031-022-00049-y)
Supplement: Supplementary file 9 — Additional file 9: Appendix 9. (a) ANCOVA: Type III Tests of Fixed effects and (b) Tukey HSD test. [file 43031_2022_49_MOESM9_ESM.docx]

Appendix 9.

(a) ANCOVA: *Type III Tests of Fixed effects and (b) Tukey HSD test*

| (a) Effect | | Sum Sq | DF | F Value | Pr > F | p < .05*  p < .001** |
| --- | --- | --- | --- | --- | --- | --- |
| (Intercept) | | 0.64 | 1 | 97.96 | 0.000 | ** |
| Pre-test | | 5.67 | 1 | 873.29 | 0.000 | ** |
| Major | | 0.05 | 1 | 7.40 | 0.007 | ** |
| Academic level | | 0.07 | 3 | 3.81 | 0.011 | * |
| Gender | | 0.04 | 1 | 6.65 | 0.010 | * |
| Major: Academic level | | 0.06 | 3 | 3.08 | 0.028 | * |
| Major: Gender | | 0.02 | 1 | 2.81 | 0.095 | ns |
| Academic level: Gender | | 0.04 | 3 | 2.19 | 0.089 | ns |
| Major: Academic level: Gender | | 0.03 | 3 | 1.68 | 0.171 | ns |
| Residuals | | 1.84 | 283 |  |  |  |
| (b) |  |  |  |  |  |  |
| Group 1 | Group 2 | diff | lwr | upr | p adj | p < .05 |
| STEM | Non-STEM | 0.03 | 0.00 | 0.05 | 0.048 | * |
| Junior | Freshman | 0.03 | -0.01 | 0.08 | 0.236 | ns |
| Senior | Freshman | 0.03 | -0.02 | 0.07 | 0.492 | ns |
| Sophomore | Freshman | 0.03 | -0.02 | 0.07 | 0.353 | ns |
| Senior | Junior | -0.01 | -0.04 | 0.02 | 0.943 | ns |
| Sophomore | Junior | 0.00 | -0.03 | 0.03 | 0.992 | ns |
| Sophomore | Senior | 0.00 | -0.03 | 0.04 | 0.993 | ns |
| Male | Female | 0.01 | -0.01 | 0.03 | 0.308 | ns |
